# The association of vitamin A, zinc and copper levels with clinical symptoms in children with autism spectrum disorders in Jilin Province, China

**DOI:** 10.1186/s12887-023-03987-2

**Published:** 2023-04-13

**Authors:** Junyan Feng, Ling Shan, Chunyue Miao, Yang Xue, Xiaojing Yue, Feiyong Jia

**Affiliations:** Department of Developmental and Behavioral Pediatrics, The First Hospital of Jilin University, Changchun, 130021 China

**Keywords:** Autism spectrum disorder, Vitamin A, Zn, Cu

## Abstract

**Background:**

This study evaluated vitamin A (VA), copper (Cu), and zinc (Zn) levels in the population with autism spectrum disorder (ASD) in Jilin Province, China. Furthermore, we examined their links to core symptoms and neurodevelopment, as well as gastrointestinal (GI) comorbidities and sleep disorders.

**Methods:**

This study included 181 children with autism and 205 typically developing (TD) children. The participants had not taken vitamin/mineral supplements in the prior three months. High-performance liquid chromatography was used to measure serum VA levels. By using inductively coupled plasma–mass spectrometry, Zn and Cu concentrations in plasma were determined. Importantly, the Childhood Autism Rating Scale, the Social Responsiveness Scale, and the Autism Behavior Checklist were used to measure core ASD symptoms. However, the Griffith Mental Development Scales-Chinese were used to measure neurodevelopment. GI comorbidities and sleep abnormalities were assessed with the 6 Item-Gastrointestinal Severity Index and Children’s Sleep Habits Questionnaire, respectively. Children with ASD with GI issues were grouped according to severity (low GI severity and high GI severity groups).

**Results:**

(i) The difference in VA, Zn, Cu levels and the Zn/Cu ratio between ASD and TD children is small. But children with ASD had lower VA levels and Zn/Cu ratio, higher Cu levels than TD children. Cu levels in children with ASD were associated with the severity of core symptoms. (ii) Children with ASD were much more likely than their TD counterparts to suffer from GI comorbidities or sleep problems. Furthermore, it was observed that high GI severity was associated with lower levels of VA, whereas low GI severity was associated with higher levels of VA. (iii) The children with ASD who had both lower VA and lower Zn/Cu ratio had more severe scores on the Autism Behavior Checklist, but not on other measures.

**Conclusion:**

Children with ASD had lower VA and Zn/Cu ratio, and higher Cu levels. Cu levels in children with ASD were weakly correlated with one subscale on social or self-help. ASD children with lower VA levels may face more serious GI comorbidities. Children with ASD combined VA-Zn/Cu lower had more severe core symptoms.

**Trial registration:**

Registration number: ChiCTR-OPC-17013502. Date of registration: 2017-11-23.

## Introduction

Autism spectrum disorder (ASD) is a neurodevelopmental disorder that is characterized by limited, repetitive activities or interests, as well as poor social communication and social interaction. In addition to these core symptoms, neurodevelopmental delays and comorbidities such as gastrointestinal and sleep issues are very common in children with ASD[1, 2]. The incidence of ASD has increased considerably during the past few decades. The overall frequency of ASD in 8-year-old children is 23.0 cases per 1,000 children (one in 44), according to the National Center for Health Statistics[3]. Although there have been impressive advances in understanding the biology of ASD, its etiology is largely unknown. In addition to oxidative stress and neurotransmitter levels, studies have shown that genetic, inflammatory, autoimmune, and environmental variables are also implicated. Minerals and vitamins, as well as other environmental elements, have been studied in connection to ASD. The impact of these nutrients on the central nervous system (CNS) has been the subject of numerous studies, the majority of which have shown a link between these nutrients (vitamins and minerals) and neurological development and cognitive performance[4, 5].

In the form of its metabolite, vitamin A (VA), also known as retinoic acid, is a necessary micronutrient for the proper functioning of different systems and biological processes. VA deficiency (VAD) is one of the most prevalent micronutrient deficiencies worldwide. VAD may be especially harmful to children and pregnant women[6]. A lack of sufficient VA in early pregnancy may harm brain development and lead to long-term or even permanent difficulties with learning and memory, as well as other aspects of cognitive function. Many studies have shown that ASD patients have lower levels of VA, which is negatively correlated with the severity of ASD core symptoms[7–10]. Tingyu Li et al. reported that the serum concentrations of VA were reduced in children with ASD in Chongqing compared with those in Hainan, and children with ASD in Chongqing had higher VA deficiency rates than those in Hainan[10]. Moreover, metal micronutrients may also have a role in the development of ASD. Cu and Zn are essential for cognitive development, appropriate brain function, and heavy metal detoxification, among other functions[11–13]. According to previous research, children with ASD are more likely to suffer from Zn lower, high Cu levels, and a low Zn/Cu ratio[12–15]. The Zn/Cu ratio has been proposed as a biomarker for ASD by certain authors[16, 17]. The Zn/Cu ratio in serum was shown to have a threshold value of 0.665, indicating an auxiliary diagnosis of autism[17]. Metal micronutrient levels in persons with ASD may also be linked to variations in location, socioeconomic determinants of health, and study design. Small sample numbers have also led to inconsistent results in some research. Min Guo et al. found that the levels of Zn in children with ASD in Hainan province were significantly lower than those in TD children, and no significant difference was found in Cu levels[8]. Si ou Li et al. reported that the mean serum Zn levels and Zn/Cu ratio were significantly lower in children with ASD in Mudanjiang city compared with normal children, whereas their serum Cu levels were significantly higher. There was a significant negative association between the Zn/Cu ratio and CARS scores[13]. However, clinical evidence on VA, Zn, and Cu levels in ASD patients is currently lacking. There are few studies on the levels of VA, Zn, and Cu in the Chinese ASD population in the northeastern region[13]. Consequently, one objective of this research was to measure VA, Zn, and Cu levels in the ASD community in Jilin Province, China, and investigate their correlation with clinical symptoms and neurodevelopmental levels.

Individuals with ASD often suffer from comorbid conditions such as gastrointestinal (GI) issues and sleep disorders, and the frequency of GI disorders and sleep irregularities is greater than children that are typically developing (TD)[18–20]. Research points out that decreased blood VA levels were observed in children with ASD with GI symptoms and sleep disorders, suggesting that lower VA levels were linked to the incidence of these comorbidities in children with ASD[9, 21]. This association between VA deficits in children with ASD, as well as their gastrointestinal and sleep disorders, must be further explored and confirmed. Although previous studies have shown that sleep length is connected to micronutrient status, sleep duration is favorably associated with Zn levels and adversely associated with Cu levels[22]. Neurotransmitter activity and circadian gene expression are two of the factors that influence the link between micronutrients and these effects[23]. However, there seems to be little research on the links between Zn and Cu levels and gastrointestinal comorbidities or sleep disorders in children with ASD. Therefore, another main objective of the current research was to investigate the links between nutritional levels (vitamin A, Zn and Cu) and gastrointestinal comorbidities and sleep disorders in children with ASD. In general, the purpose of this study was to assess VA, Zn, and Cu levels in the ASD community in Jilin Province and investigate their correlations with core ASD symptoms and neurodevelopment, as well as gastrointestinal comorbidities and sleep disorders.

## Methods

### Subjects

For this research, a total of 181 children with ASD were recruited from the Outpatient Department of Development and Behavioral Pediatrics at The First Hospital of Jilin University. Pediatricians made the diagnosis of ASD based on the DSM-5 criteria provided by the American Psychiatric Association. Among the children with ASD, the median age was 50 months. In this investigation, patients with concomitant neurological problems (such as cerebral palsy or tuberous sclerosis) or metabolic abnormalities (such as phenylketonuria) were excluded. Participants had not taken any vitamin or mineral supplements in the prior three months. Our study included 205 TD children (with a median age of 55 months) from the Changchun area’s child census, these TD children were matched with the ASD group in terms of age and sex. The TD children showed no symptoms of neuropathology or any other pathology that may affect vitamin or mineral homeostasis, and they had not taken any vitamin or mineral supplements in the prior three months.

Before including any participants, informed consent was acquired from the parents of all children who would be participating. According to the Research Ethics Committee of The First Hospital of Jilin University, this research was ethically acceptable (Approved No.20170107). This clinical study was also registered with the Chinese Clinical Trial Registry (ChiCTR) as part of the registration process (registration number: ChiCTR-OPC-17013502).

### Biochemical measurements

Three milliliters of blood were drawn from the children who participated in this research (both the ASD and TD groups). The serum and plasma were separated and kept at -20 °C until they were used for testing. The levels of VA in the serum were determined using high-performance liquid chromatography (HPLC). Moreover, the levels of Zn and Cu in plasma were determined using inductively coupled plasma–mass spectrometry (ICP–MS).

### Evaluation method

Autism Behavior Checklist (ABC), Childhood Autism Rating Scale (CARS), and Social Responsiveness Scale (SRS) were used to measure ASD symptoms in children with ASD. The ABC is a five-part behavior rating scale that is used to assess and categorize behavioral difficulties. A scale of zero to four is used to grade items, higher scores reflect a more significant issue. The CARS was developed by Schopler and Reichler et al. and is used as a diagnostic scale. It consists of 15 subscales, each of which is scored on a continuum from normal to severely abnormal. The CARS requires observation of the behavior of children with ASD in a consulting room. The SRS is a questionnaire with 65 items. Each question receives a score ranging from 1 (not true) to 4 (usually always true), with higher values indicating more social impairment associated with ASD. The reliability and validity of the ABC, CARS and SRS in the Chinese context are adequate, reflecting the scales’ utilities for the clinical diagnosis and evaluation of ASD symptoms[24, 25]. The GDS-C was used to assess the development of children with ASD. The GDS-C has six subscales: Language (A), Locomotor (B), Performance (C), Personal-Social (D), Practical Reasoning (E), and Eye-Hand Coordination (F). A general or subscale score of less than 2 SD (< 70) indicates a considerable delay, a score between 1 SD and 2 SD (70–85) indicates a slight delay, and a score equal to or more than 85 points (≥ 85) indicates no delay. The GDS-C is a popular tool in the Chinese social context and has good reliability and validity[26]. A modified GI Severity Index version was used to measure GI symptoms. In particular, only the first six components were included in the 6-GSI (abdominal pain, diarrhea, stool consistency, constipation, flatulence, and stool smell). People with scores of 3 or less were included in the low GI-severity group, while those with scores over 3 were included in the high GI severity group[27]. There are 33 questions in the CSHQ, which is a parent-report measure that is used to screen for sleep disorders. Ratings range from 1 to 3, with 3 representing frequent use (5–7 nights per week), 2 denoting infrequent use (2–4 nights per week), and 1 indicating infrequent use (0–1 night per week). The overall score is calculated by adding the frequency ratings, with higher scores indicating more sleep interruptions. The clinical threshold for sleep disorders was set at a score of 41[28].

### Statistical analysis

The SPSS Statistics 22.0 program was used to examine the data (IBM Corp., Armonk, NY). Results are expressed as means ± standard deviations (SD) or median (25th,75th percentiles) for continuous variables and percentages for categorical variables. Student’s t tests or nonparametric Mann–Whitney tests were employed to evaluate statistically significant differences in the mean values of two or more continuous variables, depending on the distribution of the studied variable. The proportions of categorical variables in two or more groups were compared using chi-square tests and nonparametric Mann–Whitney tests. To determine the importance of associated variables, Spearman’s correlation studies were performed. Multivariate logistic regression was used to detect the association between Cu levels and ASD severity. All comparisons were made using two-sided tests with a threshold of significance of 0.05. For all analyses, the null hypothesis was that there would be no differences between the research groups. Uncontrolled multiple hypothesis test is the limitation of this study.

## Results

### Demographic characteristics of the study participants

This research included 181 children with ASD and 205 TD children (Table 1). The median age of the children with ASD was 50 months. The TD children (median age of 55 months) were randomly selected and matched with the ASD group. A total of 80.67% (n = 146) of the children with ASD were male, and 19.33% (n = 35) were female, an approximately 4:1 ratio. These findings in the ASD population are in line with epidemiological data[1, 3]. A total of 75.12% (154 of the TD children) were male, and 24.88% (51 of the TD children) were female. The sex, height, weight, and diameter of the skull did not vary significantly between the two groups. Additionally, the groups did not differ in birth history factors (age of the mother at birth, age of the father at birth, gestational age, and birth weight).

**Table 1:** Demographic characteristics of ASD and TD children

Parameter	ASD children (n = 181)	TD children (n = 205)	Z/t/χ2	P
Age (month)	50.0 (41.3, 62.0)	55.0 (40.0, 68.0)	-0.981	0.327
Height (cm)	106.9 (100.0, 116.4)	105.0 (96.1, 115.0)	-1.788	0.074
Body weight (kg)	18.0 (16.0, 22.5)	18.3 (15.4, 23.2)	-0.318	0.751
Head circumference (cm)	50.5 (49.5, 51.9)	51.0 (49.0, 52.0)	-0.509	0.611
Sex				
Male (%)	146.0 (80.7)	154.0 (75.1)	1.704	0.192
Female (%)	35.0 (19.3)	51.0 (24.9)		
Age of the mother at birth (years)	27.0 (24.0, 32.0)	29.0 (26.5, 31.5)	0.928	0.353
Age of the father at birth (years)	30.0 (26.0, 34.0)	30.0 (28.0, 33.5)	0.735	0.463
Gestational age (weeks)	38.1 ± 1.6	38.8 ± 1.2	0.832	0.415
Birth weight (kg)	3.6 (3.1, 4.0)	3.4 (3.0,3.8)	1.168	0.243

Data are expressed as means ± SD or median (25th,75th percentiles) or percentages for categorical variables

### ASD children have lower VA and higher Cu compared to TD children

**Fig. 1:**
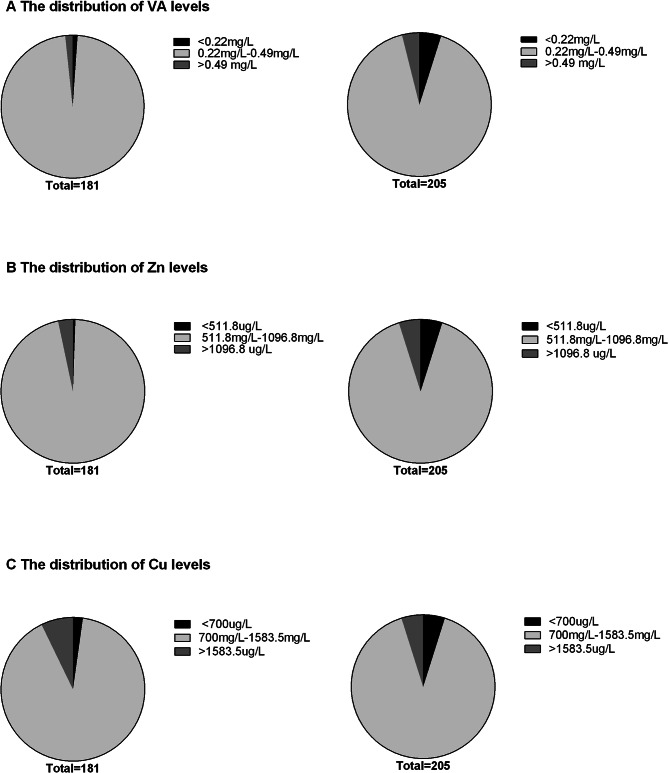
The proportion of VA, Zn and Cu abnormalities in ASD patients and TD children. The values on the left are for ASD, and on the right are for TD.

We established a 5-95% reference range of VA/Zn/Cu level for children with TD. It can be seen from Fig. 1 that the distribution difference of VA, Zn and Cu levels between the two groups is small. Next, we compare the levels of VA, Zn, Cu levels and the Zn/Cu ratio. There was a statistically significant difference about the serum VA concentrations [0.34 (0.3, 0.39) vs. 0.37 (0.31, 0.41), P = 0.026] and the Zn/Cu ratio [0.64(0.52, 0.75) vs. 0.74(0.59, 0.88), P = 0.000], which were considerably lower in the 181 children with ASD than in the 205 TD children. Cu levels were considerably higher in children with ASD than in TD children [1202.5 (1040,1390) vs. 1070 (929.5,1250), P = 0.000]. Zn levels were found to be lower in children with ASD than in TD children, although no statistically significant difference between the two groups was found (Table 2).

**Table 2:** The VA, Zn, Cu levels and Zn/Cu ratios in ASD patients and TD children

Parameter	ASD children (n = 181)	TD children (n = 205)	Z	P
VA (mg/L)	0.34 (0.30, 0.39)	0.37 (0.31, 0.41)	-2.219	0.026*
Zn (ug/L)	755.0 (702.0, 867.0)	790.0(674.5, 918.5)	-0.817	0.414
Cu (ug/L)	1202.5 (1040.0, 1390.0)	1070.0 (929.5, 1250.0)	-5.065	0.000*
Zn/Cu	0.64 (0.52, 0.75)	0.74 (0.59, 0.88)	-4.272	0.000*

Data are expressed as median (25th,75th percentiles).*P < 0.05

### Cu levels correlate with core symptoms in children with ASD

We examined the relationships between ASD symptom scores (total and subscale scores of the ABC, CARS, SRS, and GDS-C) and VA and Cu levels as well as Zn/Cu ratios in children with ASD. The Cu level was positively correlated with the score on the social or self-help subscale of the ABC (r = 0.213, P = 0.005). No significant relationship between VA, Zn/Cu levels and core ASD symptoms or neurodevelopmental level was found (Table 3). Multivariate logistic regression analysis showed that Cu level was significantly associated with the score on the social or self-help subscale of the ABC, after adjusting for VA and Zn (B = 0.004, T = 2.875, P = 0.005).

**Table 3:** Correlations of VA, Cu levels and Zn/Cu ratios with clinical symptoms in ASD children

	VA	Cu	Zn/Cu
Variable	R		
ABC			
Total score	-0.094	0.041	-0.045
body and object use subscale	-0.080	0.085	-0.179
sensory subscale	-0.142	0.134	-0.148
social or self-help subscale	-0.107	0.213*	-0.161
language subscale	-0.075	-0.109	0.100
social skills subscale	-0.019	0.132	-0.073
Total CARS score	-0.115	0.117	-0.115
SRS			
Total score	-0.016	0.092	0.026
social awareness subscale	-0.025	0.104	0.051
social cognition subscale	0.081	0.024	0.139
social communication subscale	-0.086	0.081	-0.015
social motivation subscale	-0.082	0.131	0.002
autistic mannerisms subscale	0.045	0.06	0.030
GDS-C-A	0.138	-0.129	0.056
GDS -C-B	0.078	-0.127	0.074
GDS-C-C	0.012	-0.212	0.073
GDS-C-D	-0.008	-0.160	0.022
GDS-C-E	0.122	-0.230	0.093
GDS-C-F	-0.102	-0.200	0.122

GDS-C-A: locomotor subscale of the GDS-C, GDS-C-B: personal and social skills subscale of the GDS-C, GDS-C-C: hearing and language subscale of the GDS-C, GDS-C-D: hand-eye coordination subscale of the GDS-C, GDS-C-E: performance subscale of the GDS-C. *P < 0.01

### The levels of VA, Zn, and Cu, as well as the Zn/Cu ratio in children with ASD with or without GI comorbidities and sleep abnormalities

All participants’ 6-GSI and CSHQ scores were acquired. The proportion of children with GI comorbidities (60.22% vs. 25.85%, p = 0.000) and sleep abnormalities (90.06% vs. 35.6%, p = 0.000) in the ASD group was significantly higher than that in the TD group (Table 4). We further divided the children with ASD into two groups according to scores on the 6-GSI: the low GI-severity group (defined as having 6-GSI scores of 3 or less) and the high GI-severity group (defined as having 6-GSI scores above 3). VA levels in the low GI-severity group were higher than those in the high GI-severity group [(0.37 (0.32, 0.42) vs. 0.33 (0.29, 0.365), P < 0.05, Table 5)]. We also divided the children with ASD into two groups according to scores on the CSHQ: the no-sleep-problems group (defined as having CSHQ scores ≤ 41) and the sleep-problems group (defined as having CSHQ scores > 41). No significant difference was found in the levels of VA, Zn, or Cu or the Zn/Cu ratios in children with ASD with or without sleep abnormalities.

**Table 4:** The combined percentage of ASD and TD children with GI comorbidities and sleep abnormalities

	low GI-severity group (≤ 3)	High GI-severity group (> 3)	No sleep problems (≤ 41)	Sleep problems (> 41)
ASD children (n = 181)	72.0 (39.8%)	109.0 (60.2%)	18.0 (9.9%)	163.0 (90.0%)
TD children (n = 205)	152.0 (74.2%)	53.0 (25.9%)	132.0 (64.4%)	73.0 (35.6%)
χ2	46.617		117.650	
P	0.000*		0.000*	

Data are expressed as percentages for categorical variables. *P < 0.05

**Table 5:** The levels of VA, Zn, and Cu, as well as Zn/Cu ratios in ASD children with or without GI comorbidities and sleep abnormalities

	low GI-severity group(n = 72)	High GI-severity group(n = 109)	P	Uncombined sleep abnormalities group(n = 18)	Combined sleep abnormalities group(n = 163)	P
VA (mg/L)	0.37 (0.32, 0.42)	0.33 (0.29, 0.37)	0.000*	0.34 (0.30, 0.38)	0.34 (0.31, 0.42)	0.566
Zn (ug/L)	757.0(702.0, 883.0)	758.0 (704.5, 863.5)	0.940	755.0 (699.0, 867.0)	781.5 (738.0, 867.8)	0.576
Cu (ug/L)	1213.0 (1029.0, 1380.0)	1188.0 (1043.0, 1416.8)	0.673	1202.0(1037.0,1407.0)	1172.0 (1048.0, 1290.0)	0.150
Zn/Cu ratio	0.65 (0.53, 0.85)	0.63(0.55, 0.73)	0.480	0.62 (0.53, 0.77)	0.69 (0.59, 0.75)	0.203

Data are expressed as median (25th,75th percentiles).*P < 0.05

### Effects of VA levels and Zn/Cu ratios on core symptoms, neurodevelopmental levels, GI comorbidities and sleep abnormalities in children with ASD

We used the median VA level (0.34) and Zn/Cu ratio (0.643) as cutoff values to divide the children with ASD into four groups: Combined VA-Zn/Cu lower group (VA level < 0.34 and Zn/Cu ratio < 0.643), the lower VA level group (VA level < 0.34 but Zn/Cu ratio > 0.643) ,the lower Zn/Cu ratio group (Zn/Cu ratio < 0.643 but VA level > 0.34) and combined VA-Zn/Cu higher group (VA level ≥0.34 and ≥Zn/Cu ratio 0.643). Compared to the other three groups, combined VA-Zn/Cu lower group had higher total and subscale scores on the ABC, as well as higher scores on body and object use subscale, the sensory subscale, social or self-help subscale, and language subscale (all p < 0.05, Table 6).

**Table 6:** Effect of VA levels and Zn/Cu ratios on scores on the ABC, CARS, SRS, GDS-C, 6-GSI, and CSHQ in ASD children

	Combined VA-Zn/Cu lower group (N = 42)	Lower VA group (N = 39)	Lower Zn/Cu ratio group (N = 48)	Combined VA-Zn/Cu higher group (N = 52)	P
ABC					
Total score	56.0 (45.0, 72.0)	42.0(34.0, 56.0)a	40.0 (23.0, 57.0)b	44.0(25.0,59.0)c	0.001*
body and object use subscale	11.0 (5.5, 14.0)	7.0(4.0, 11.0) a	7.0 (3.0, 13.0)b	6.0(2.0,10.0)c	0.018*
sensory subscale	10.0 (5.5, 14.0)	7.0(2.0, 12.0)a	6.0 (3.0, 9.0)b	6.0(3.0,10.0)c	0.019*
social or self-help subscale	11.0 (7.0, 14.0)	8.0(5.0, 11.0)a	9.0 (6.0, 12.0)b	8.0(5.0,11.0)c	0.014*
language subscale	13.0 (11.0, 16.0)	11.0 (7.0, 13.0)a	9 0.0(5.8, 12.0)b	9.0(5.3,13.0)c	0.003*
social skills subscale	13.0 (9.8, 17.0)	10.0 (8.0, 15.0)	13.0 (8.0, 16.0)	12.0(5.0,17.0)	0.565
Total CARS score	31.0 (29.0,34.0)	28.5 (26.0, 32.0)	30.0 (26.0, 32.0)	29.0(26.0,33.0)	0.118
SRS					
Total score	87.0 (79.0, 96.0)	91.0 (70.0, 109.0)	89.0 (66.0, 112.0)	85.0(64.0,107.0)	0.905
social awareness subscale	13.0 (11.0, 14.0)	13.0 (12.0, 17.0)	13.0 (6.0, 17.0)	12.0(10.0,15.0)	0.190
social cognition subscale	18.0 (13.0, 20.0)	18.0 (13.0, 22.0)	17.0 (11.0, 21.0)	17.0(16.0,21.0)	0.697
social communication subscale	33.0 (28.0, 40.0)	31.0 (27.0, 40.0)	32.0 (23.0, 40.0)	29.0(24.0,39.0)	0.851
social motivation subscale	14.0 (12.0, 15.0)	13.0 (9.0, 16.0)	11.0 (7.5,14.0)	14.0(8.5,16.5)	0.274
autistic mannerisms subscale	13.0 (8.0, 15.0)	12.0 (5.0, 17.0)	11.0 (4.5, 18.0)	14.0(5.5,18.0)	0.969
GDS-C-A	74.5 (58.2, 86.5)	67.5 (56.0, 78.3)	69.0 (61.0, 94.0)	74.5(62.3,91.5)	0.428
GDS -C-B	57.0 (48.0, 73.0)	56.5 (42.8, 80.0)	62.0 (52.0, 73.5)	57.0(47.5,74.0)	0.916
GDS-C-C	48.0 (32.0, 72.0)	52.5 (32.8, 70.0)	46.0 (34.0, 70.3)	49.0(38.0,65.3)	0.999
GDS-C-D	61.0 (47.0, 74.0)	62.0 (39.8, 84.5)	62.0 (43.5, 79.8)	62.0(50.0,74.5)	0.997
GDS-C-E	61.0 (46.0, 80.3)	68.5 (39.8, 76.0)	68.0 (51.0, 81.0)	65.0(51.0,81.5)	0.776
GDS-C-F	64.0 (52.0, 87.0)	68.0 (51.0, 97.0)	57.0 (39.0, 77.0)	65.5(50.8,79.0)	0.591
6-GSI (>3)	30.0(71.4%)	27.0(69.2%)	25.0(52%)	27.0(42.3%)	0.096
CSHQ	48.0 (44.8, 52.3)	48.0 (42.0, 51.0)	49.5 (45.3, 53.0)	48.0(44.3,54.0)	0.261

Data are expressed as median (25th,75th percentiles) or percentages for categorical variables. *P < 0.05.GDS-C-A: locomotor subscale of the GDS-C, GDS-C-B: personal and social skills subscale of the GDS-C, GDS-C-C: language subscale of the GDS-C, GDS-C-D: hand-eye coordination subscale of the GDS-C, GDS-C-E: performance subscale of the GDS-C. GDS-C-F: practical reasoning subscale of the GDS-C. P-value is for a comparison of the Combined VA-Zn/Cu lower group vs. the other 3 groups. a: combined VA-Zn/Cu lower group vs. lower VA group (P < 0.05), b: combined VA-Zn/Cu lower group vs. lower Zn/Cu ratio group (P < 0.05), c: combined VA-Zn/Cu lower group vs. combined VA-Zn/Cu higher group (P < 0.05)

## Discussion

The primary focus of this research was to assess VA, Zn, and Cu levels in children with ASD and their association with core ASD symptoms and neurodevelopment, as well as gastrointestinal comorbidities and sleep disorders. The following are the three most important conclusions of this study: (i) Children with ASD had lower VA and higher Cu levels, as well as a lower Zn/Cu ratio than TD children, and The Cu levels may be positively associated with core ASD symptoms. (ii) Children with ASD had more GI comorbidities and sleep abnormalities, and the levels of VA in these children were associated with GI comorbidities. (iii) Children with ASD combined VA-Zn/Cu lower have aggravated core ASD symptoms.

VA is a vitamin that is required for proper development and function of the brain[29]. Serum retinol concentrations ≥ 0.3 mg/L were defined as VA normal, < 0.3 mg/L and ≥ 0.2 mg/L were defined as marginal VA deficiency, < 0.2 mg/L were defined as VA deficiency, and < 0.1 mg/L were defined as severe vitamin A deficiency[9]. The level of VA in ASD children is controversial. Adams et al. show that VA has no significant difference between ASD children and healthy control[30],but some studies found that VA is lower in ASD than healthy control[7–10].Other studies have shown different outcomes. Among American children with ASD between the ages of 4 and 8 years, Hyman discovered that they had lower VA levels, while various age groups had higher VA levels[31]. In this investigation, the serum VA concentration in children with ASD was shown to be considerably lower than that in TD children. According to earlier studies[7–10], this outcome is consistent. This discrepancy might be explained by the differences in eating preferences across various locations, as well as the high food selectivity of certain children with ASD. Several studies have shown that VA lower may be linked to the genesis of ASD. Several mechanisms have been proposed, including (1) VA may impact the nervous system by changing synapses, the hippocampus, and other structures in the brain. (2) VA may impact the digestive system by regulating gut flora and lymphoid tissue. (3) VAD may decrease oxytocin release by controlling the expression of cluster difference 38 (CD-38). (4) VA may reduce ASD-like symptoms by regulating 5-hydroxytryptamine (5-HT) levels[29]. However, we did not discover a link between VA level and the core symptoms and neurodevelopment. The relationship between VA and children with ASD needs further study.

Cu and Zn, which are essential for the development of the CNS, are involved in a complex regulatory network that is responsible for maintaining CNS homeostasis from the earliest stages of life. Several studies have shown that the metabolism of Cu and Zn is disrupted in ASD. Zn deficiency, high Cu levels, and a poor Zn/Cu ratio are all prevalent in children with ASD[12–14, 17]. In our investigation, we discovered that there was no statistically significant difference in Zn levels, but Zn levels tended to be lower in children with ASD than in TD children. Nevertheless, the researchers discovered that children with ASD had higher Cu levels and a lower Zn/Cu ratio than TD children. The concentration of Cu in children with ASD, as well as the Zn/Cu ratio, were shown to be connected with the severity of their symptoms. Zn helps to maintain a healthy equilibrium with Cu in the blood, where variations in the levels of these two trace elements are inversely proportional. Lower Zn/Cu ratios may indicate a Zn deficit in the body as a whole or the buildup of toxic metals that are antagonistic to Zn. Bjørklund et al. hypothesized that Zn deficiency in ASD is primarily caused by other metabolic disturbances that result in increased oxidative stress, such as increased mitochondrial ROS production, whereas Cu excess occurs as a result of both increased oxidative stress and abnormally elevated cytokine levels (which increase the production of ceruloplasmin in the liver), which may be caused by intestinal dysfunction and changes in the intestinal microflora. This does not rule out the potential of additional hereditary biochemical disorders affecting Cu metabolism directly[11]. Several lines of evidence indicate that Zn and Cu may play a role in the GABAergic system and that low Zn and high Cu levels may influence GABA production or membrane transport, eventually leading to a reduction in GABA concentrations in the synaptic cleft[32, 33]. Additionally, Zn affects the glutamatergic system[32]. It has been estimated that the synaptic vesicles of glutamatergic neurons contain approximately 10% of the total Zn in the brain. In GABA and glutamate modulation, Zn is connected with anxiolytic action, modifying GABAergic inhibition and seizure susceptibility, and is notably associated with anxiolytic function. Furthermore, Zn lower is related to GABAergic dysfunction[33]. Cu is a powerful inhibitor of GABA-evoked responses, primarily in Purkinje cells. As a result, Zn and Cu may interact with one another and the GABAA receptor complex to influence synaptic transmission [32, 33]. Additionally, Cu is a cofactor essential for the function of dopamine-β-hydroxylase[34], a neurotransmitter-synthesizing enzyme that converts dopamine to norepinephrine. A high Cu level may also be connected with the elevated norepinephrine levels reported in children with ASD. Cu poisoning has a significant impact on the brain, depending on the degree of toxicity and an individual’s sensitivity to Cu, toxic amounts of Cu may have a mild to severe impact on the brain[35]. The possible neurotoxic consequences of excess Cu include the perceptual problems observed by those suffering from schizophrenia, sadness, irritability, fear, anxiousness, and learning and behavioral difficulties[35].

The current research also revealed that the frequency of GI comorbidities and sleep disorders was considerably greater in children with ASD than in children without ASD, which is similar to results from previous studies[18–20]. VA levels were higher in the low GI severity group than in the high GI severity group, which was consistent with prior research[10, 36]. The mechanism behind GI problems in children with ASD, however, remains unknown. VA levels have been linked to gastrointestinal function in several studies. VA might lead to GI comorbidities, but GI tract is a complex organ that provides micronutrients extracted from the ingested food, GI comorbidities could also lead to lower VA. VA supplementation alleviates diarrhea and reduces intestinal damage[37–39]. Tingyu Li and colleagues developed valproic acid-induced ASD rat models with varying VA concentrations. ASD model rats with gestational VAD demonstrated severe autism-like behaviors (especially social deficits), worsened GI motility and enteric nervous system impairments compared to ASD model rats with normal VA levels. The increased enteric nervous system dysplasia may be caused by decreased expression of retinoic acid receptor alpha and modulation of the Ret signaling pathway as a result of VA binding to the promoter region of the Ret gene. Supplementation with VA reduced autism-like symptoms and enteric nervous system dysplasia in an ASD rat model with VAD[36]. Although previous studies have shown that sleep duration is positively correlated with zinc level and negatively correlated with copper level[22]. No significant difference was found in the levels of VA, Zn, or Cu, or the Zn/Cu ratios in ASD children with or without sleep abnormalities in our study. We only analyzed the total score of CSCQ, without further analysis from sleep habits, sleep duration and other aspects. In addition, the mismatched number of cases between combined sleep abnormalities group (n = 163) or Uncombined sleep abnormalities group (n = 18) may also be a reason. Notably, we found that ASD children combined VA-Zn/Cu lower had more severe core symptoms and a higher proportion of GI comorbidities than those with either low VA levels or low Zn/Cu ratios. Zinc may be required for vitamin A mobilization from its storage site in the liver. Zinc-deficient animals have been reported to have lower circulating vitamin A mobilization from the liver, perhaps due to impaired synthesis of the plasma retinol binding protein [40]. In addition, Rachman et al. investigated the consequences of a copper-deficient diet on liver and blood vitamin A storage in Wistar rats. They have observed in the liver of the rats fed a copper-deficient diet a significantly higher mean level of retinyl esters and retinol compared to the mean concentration of the retinyl esters and retinol in controls [41]. Therefore, we speculate that the combined vitamin A-Zn/Cu lower may have synergistic effects and further aggravate the impact on children with ASD. Therefore, it is critical to conduct timely, intensive, and evidence-based nutritional interventions. We hope these data can guide the precise treatment of patients with specific types of ASD. The results will help to inform the nutritional management of children with ASD.

## Limitation of the study

However, there are some limitations to the current study. We did not screen for the most common singe-gene syndromic ASD, such as fragile X syndrome. Screening and ruling out FXS would decrease the enormous heterogeneity that exists in idiopathic ASD. Information on the diets and food choices of children with ASD was not collected. In addition, we only evaluated VA, Zn, and Cu levels in children with ASD, we did not apply nutritional interventions and assess changes in core symptoms, neurodevelopment, or GI comorbidities.

## Conclusion

Children with ASD had lower VA levels and Zn/Cu ratio, and higher Cu levels. Cu levels in children with ASD were weakly correlated with one subscale on social or self-help. Notably, we found that children with ASD combined VA-Zn/Cu lower had more severe core symptoms. Children with ASD had more gastrointestinal and sleep disorders than TD children. ASD children with lower VA levels may face more serious GI comorbidities.

## Data Availability

The datasets used and/or analyzed during the current study are available from the corresponding author upon reasonable request.

## Abbreviations

VA: vitamin A
Cu: copper
Zn: zinc
GI: gastrointestinal
TD: typically developing
CNS: central nervous system
VAD: VA deficiency
ABC: Autism Behavior Checklist
CSHQ: Children’s Sleeping Habits Questionnaire
SRS: Social Responsiveness Scale
GDS-C: Griffith Mental Development Scales-Chinese
CARS: Childhood Autism Rating Scale
6-GSI: Gastrointestinal Severity Index
HPLC: high-performance liquid chromatography
ICP–MS: inductively coupled plasma–mass spectrometry
5-HT: 5-hydroxytryptamine
